# Integrating Artificial Intelligence, Circulating Tumor DNA, and Real-World Evidence to Optimize Hematologic Clinical Trials: Toward Adaptive and Learning Trial Designs

**DOI:** 10.3390/cancers18071173

**Published:** 2026-04-06

**Authors:** Abdurraouf Mokhtar Mahmoud, Jasmitaben Prakashbhai Touti, Syed Rubina Zaidi, Ahad Ahmed Kodipad, Clara Deambrogi

**Affiliations:** 1 Division of Hematology, Department of Translational Medicine, University of Eastern Piedmont, 28100 Novara, Italy; 2 Hematology Clinical Trial Unit, Azienda Ospedaliero-Universitaria Maggiore della Carità, Via Solaroli 17, 28100 Novara, Italy; 3 Department of Pediatric Abdominal Transplantation and Hepatopancratobilary Surgery, Nationwide Children’s Hospital, Columbus, OH 43205, USA

**Keywords:** artificial intelligence, hematologic malignancies, circulating tumor DNA, real-world evidence, clinical trial design, precision medicine, adaptive trials

## Abstract

The integration of emerging technologies and real-world data is transforming the landscape of hematologic clinical trials. Artificial intelligence (AI) offers remarkable capabilities for predictive modeling, patient stratification, and adaptive trial design, while circulating tumor DNA (ctDNA) provides a minimally invasive biomarker for disease monitoring, the early detection of relapse, and treatment response assessment. Concurrently, real-world evidence (RWE) complements traditional clinical trial data by capturing treatment effectiveness, safety, and patient outcomes in broader, heterogeneous populations. This review examines the synergistic potential of AI, ctDNA, and RWE to optimize trial design and decision-making in hematologic malignancies. We discuss methodological innovations, including AI-driven patient selection, ctDNA-guided adaptive interventions, and the incorporation of RWE for external control arms and post-marketing surveillance. Key challenges, such as data standardization, regulatory considerations, and ethical implications, are also addressed. By integrating these advanced tools, clinical trials in hematology can achieve greater efficiency, precision, and translatability, ultimately accelerating the development of personalized therapies and improving patient outcomes.

## Simple Summary

Hematologic malignancies are complex diseases that often require carefully designed clinical trials to test new treatments. However, traditional clinical trials can be slow, expensive, and may not fully represent the diversity of patients seen in everyday clinical practice. New approaches are therefore needed to improve how these studies are conducted. In recent years, three important tools have emerged: artificial intelligence (AI), circulating tumor DNA (ctDNA), and real-world evidence (RWE). AI can help analyze large amounts of data to identify suitable patients and support more flexible trial designs. ctDNA, a blood-based biomarker, allows monitoring the disease progression and treatment response in a minimally invasive way. RWE, based on data collected from routine clinical care, provides insights into how treatments perform in real-life settings. This review explains how combining these tools can improve the design and efficiency of clinical trials in hematologic malignancies. Their integration has the potential to accelerate the development of more personalized therapies and ultimately improve patient outcomes.

## 1. Introduction

Hematological malignancies represent the fifth most common group of cancers and are characterized by considerable molecular and clinical heterogeneity, which limits the sensitivity and generalizability of traditional, rigid trial designs. However, the integration of artificial intelligence (AI), circulating tumor DNA (ctDNA), and real-word evidence (RWE) is now creating new opportunities as “learning trials”. In this model, AI functions as the analytic core, employing machine learning (ML) and natural language processing (NLP) to mine high-dimensional electronic health records (EHR) and multi-omics data, efficiently phenotyping patients and identifying a molecular subtype suitable for highly targeted studies [1,2,3,4,5]. Simultaneously, circulating tumor DNA (ctDNA) serves as a minimally invasive “liquid biopsy” and dynamic biomarker of minimal residual disease (MRD), capable of detecting molecular relapse and treatment resistance months before conventional imaging or clinical progression, and increasingly being incorporated into adaptive and dose-finding trial designs to shorten study duration and optimize dosing decisions [6,7,8]. RWE derived from routine clinical practice, when standardized and analyzed using AI, can generate external or synthetic control arms (SCAs). This approach enhances trial feasibility and ethical acceptability for rare, relapsed, or molecularly defined patient populations. It also improves generalizability compared to the restrictive eligibility criteria of traditional randomized controlled trials (RCTs) [1,3,5,9]. Taken together, RWE provides essential context by using data from routine clinical practice to generate SCAs, thereby, reducing the ethical and logistical burden of recruiting placebo groups in rare or relapsed populations.

The integrated framework aims to replace rigid, uniform trial structures with hybrid adaptive designs. In these designs, AI-derived RWE refines eligibility criteria and cohort selection, while ctDNA provides dynamic surrogate endpoints and MRD assessments. Traditional clinical endpoints maintain the rigor necessary for regulatory validation [10,11]. Emerging data from AI-facilitated, biomarker-adaptive, and hybrid RWE-RCT approaches suggest a potential reduction in development timelines. These gains arise from expedited surrogate endpoints combined with external or synthetic controls, without compromising statistical validity or regulatory compliance [10,12,13,14]. This effectively reframes drug development from a static, protocol constrained experiment into a continuously learning clinical ecosystem that iteratively updates trial conduct and therapeutic decisions in parallel with each patient’s evolving disease course [3,10,15]. Despite the rapid evolution of AI, ctDNA, and RWE, these domains are often explored in isolation. However, their true transformative potential emerges when they are integrated within a unified clinical trial framework. AI provides the computational infrastructure for predictive modeling and adaptive decision-making, ctDNA enables real-time molecular monitoring of disease dynamics, and RWE ensures external validity by capturing outcomes across diverse patient populations. Together, these technologies enable the development of adaptive, continuously learning clinical trial systems that bridge the gap between controlled experimental settings and real-world clinical practice. Therefore, this review aims not only to summarize these advances individually but to highlight their synergistic integration as a foundation for next-generation hematologic clinical trials.

## 2. Challenges in Conventional Hematology Clinical Trials

### 2.1. Diseases and Trial Design Complexity

The field of hematology has made significant advances in the biological understanding and therapeutic management of hematological malignancies; however, traditional clinical trial frameworks often fail to keep pace with this rapid progress. Trial methodologies are challenged by marked disease heterogeneity, multiple prior therapeutic exposures, and growing complexity of targeted and immunotherapeutic agents; collectively, these factors necessitate larger patient cohorts, more granular outcome measures, and longer study durations to detect clinically meaningful treatment effects [16].

### 2.2. Recruitment, Representativeness and Equity

Restrictive eligibility criteria, demanding visit schedules, and the centralization of trials in specialized academic centers limit recruitment and retention, particularly among older adults, patients with comorbidities, and individuals from geographically rural or socioeconomically disadvantaged communities. These limitations reduce both statistical power and generalizability of trial findings [16,17,18]. Additional barriers, including travel demands, caregiving responsibilities, insurance constraints, and the centralization of hematologic malignancy and cellular therapy trials at comprehensive cancer centers further exacerbate enrolment inequities [16,18].

### 2.3. Outcome Selection, Reporting, and Patient-Centredness

Selective outcome reporting and underreporting of patient-reported outcomes (PROs) further constrain the interpretability and patient-centeredness of hematology trials. Systematic reviews have documented frequent discrepancies between registered and published endpoints in hematology RCTs, including, the addition, omission, or demotion of primary outcomes that often favor statistically significant result [19]. In phase III trials of hematologic malignancies, although nearly three-quarters prespecify PROs, only a minority report them in primary publications, typically when findings are neutral or favorable [17,19].

### 2.4. Regulatory, and Modality Specific Challenges

Traditional clinical trial frameworks struggle to incorporate innovative therapies such as cellular treatments, NK-cell and CAR-T products, and therapeutic vaccines into regulatory systems originally designed for small molecule chemotherapies and conventional biologics [20,21,22]. This creates uncertainty in endpoint selection, safety monitoring, and long-term follow-up requirements [20,22,23]. As a result, interest is rapidly increasing in decentralized clinical trials (DCTs) and hybrid designs that incorporate telemedicine, home-based procedures, wearable technologies, and community-based sites to reduce travel burden, expand access, and better align trial conduct with real-world practice. Evidence from cross-disease evaluations of DCTs suggests that, when carefully implemented, decentralized methodologies can enhance accessibility, participant diversity, and patient engagement. However, these approaches also introduce challenges related to data reliability, digital literacy gaps, data privacy and cybersecurity risks, and equity considerations, all of which require targeted mitigation strategies to prevent the widening of existing disparities [17,24,25,26,27,28]. To maximize inclusivity, recent frameworks emphasize purposeful engagement of patients and individuals with lived experience in trial design, the use of digital tools to support community-based recruitment and bidirectional communication, and the implementation of deliberate diversity, equity, and inclusion (DEI) strategies tailored to underserved populations [17,25,28]. Moreover, the regulatory pathway for this AI-integrated framework remains unclear, especially for dynamic or continuously learning decision support models. Leading agencies such as the FDA and EMA have begun issuing high-level guidance and risk-based principles for AI/ML in drug development, clinical trials, and regulatory workflows, yet these fall short of establishing a standardized, comprehensive approval process for adaptive AI systems [29]. Dynamic AI systems, including adaptive models and in silico “digital twin” approaches, exacerbate these uncertainties by conflicting with conventional one-time validation paradigms and posing challenges in managing continuous learning, dataset shift, bias, and performance drift that should be managed within a compliant framework [30,31].

Finally, the integration of RWE derived from registries and routine clinical practice into the evidence hierarchy provides an important complement to traditional RCTs, enabling assessment of effectiveness, toxicity, and access across broader, more diverse hematology populations than can feasibly be enrolled on protocolized studies [24,25,32]. When combined with decentralized and adaptive trial designs, RWE-informed eligibility criteria, and robust PRO and equity frameworks, these innovations offer a pathway to address the inherent limitations of conventional hematology trials and to support more representative, patient-centered, and methodologically rigorous evaluation of emerging therapies [33,34].

## 3. Artificial Intelligence in Hematological Malignancies

**Figure 1:**
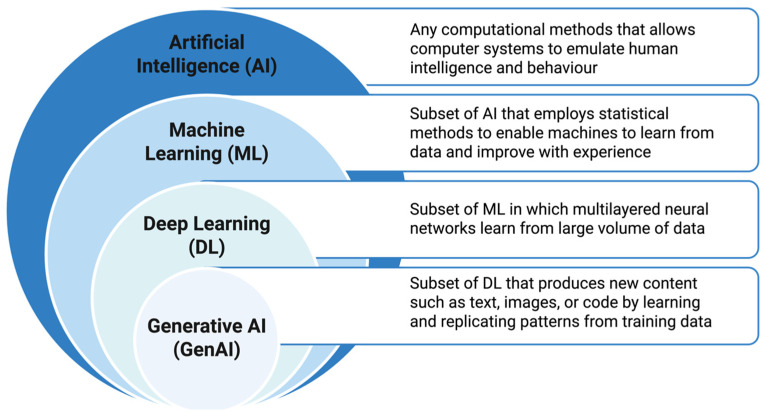
Conceptual hierarchy of artificial intelligence methodologies. Artificial intelligence (AI) denotes the broad discipline concerned with designing systems capable of tasks associated with human intelligence. Machine learning (ML) is a subfield of AI that relies on data-driven statistical methods to enable systems to learn and improve with experience. Deep learning (DL) is a specialized branch of ML based on multilayer artificial neural networks that learn hierarchical representations from large-scale data. Generative artificial intelligence (GenAI) and deep learning (DL) are overlapping but distinct domains. While many modern GenAI approaches, including large language models and diffusion models, are based on deep learning architectures, generative modeling has historically also included probabilistic and Bayesian methods that are not dependent on DL. Therefore, GenAI and DL should be considered intersecting fields rather than a strictly hierarchical relationship (created in BioRender. t, J. Mahmoud. A.M. (2026) https://BioRender.com/4hy22h1, accessed on 26 January 2026).

Artificial intelligence (AI) is increasingly reshaping the field of hematology, with a notable acceleration in both research output and clinical-oriented applications since approximately 2019. Contemporary AI methodologies including machine learning and deep learning approaches (Figure 1), are being incorporated across multiple aspects of hematologic disease management, including early disease detection, diagnostic classification, treatment planning and personalization, prognostic risk stratification, patient monitoring and management, and clinical decision support systems [35,36,37]. The expansion of AI has been driven by advances in ML and deep learning (DL) methodologies, alongside the increasing availability of large-scale datasets from digital pathology, flow cytometry, and EHRs. The COVID-19 pandemic further accelerated this digital transformation, reinforcing the central role of AI applications in the future hematology practice [38].

Hematological malignancies represent a broad and heterogeneous group of neoplasms, including leukemia, lymphoma, and multiple myeloma, and are characterized by complex underlying biology and highly heterogeneous clinical behaviors [39,40,41,42]. This diversity, reflected in distinct genetic, epigenetic, and microenvironmental characteristics, translates into widely variable responses to antineoplastic therapies [43]. Historically, diagnostic evaluation and treatment selection have relied predominantly on histopathologic assessment and clinical judgment, resulting in relatively standardized approaches despite substantial variability in clinical outcomes [44,45].

**Figure 2:**
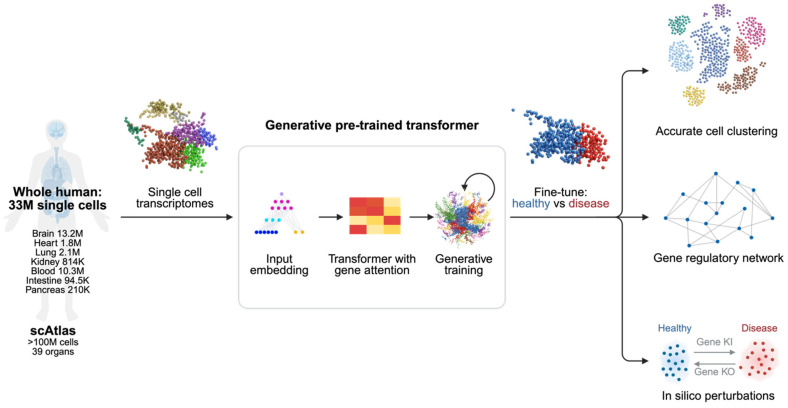
Schematic of a machine learning-based pipeline for identifying disease-associated targets from single-cell transcriptomic data. Single-cell RNA sequencing profiles are transformed into low-dimensional representations using neural networks or graph-based models to capture cellular heterogeneity and relationships. These embeddings enable the separation of healthy and diseased cell states, the identification of disease-relevant cell populations, and the inference of gene or cell interaction networks, facilitating the prioritization of candidate therapeutic targets (created in BioRender. t, J. Mahmoud. A.M. (2026) https://BioRender.com/la6ynnx, accessed on 26 January 2026).

However, the advent of AI technologies including ML algorithms, NLP, and advanced imaging analytics, has the potential to enhance precision medicine in this domain. By leveraging rich multi-omics datasets (including genomics, transcriptomics, and proteomics), AI methods enable the identification of novel molecular biomarkers, support the prediction of disease evolution, and guide the personalization of treatment decisions for individual patient profiles (Figure 2) [46,47]. The field has demonstrated remarkable progress, with measurable improvements in diagnostic accuracy, risk stratification, and patient outcomes across multiple hematological malignancies.

For instance, the integration of AI into predictive modeling can markedly improve risk stratification, enabling clinicians to implement personalized treatment strategies guided by patient-specific genomic and phenotypic characteristics, ultimately improving survival outcomes and quality of life [48]. Moreover, generative AI holds promise for accelerating the development of novel therapeutic strategies by simulating treatment responses and optimizing drug combinations, proving especially advantageous for complex conditions like acute myeloid leukemia (AML) [49]. However, the effective implementation of these advanced technologies depends on addressing challenges related to data quality, algorithmic bias, and interpretability, necessitating a collaborative approach among healthcare professionals, data scientists, and regulatory bodies to ensure equitable access and effective integration into clinical practice [37,50].

As summarized in Table 1, contemporary AI-based investigations demonstrate a wide range of applications across hematologic malignancies. In leukemia, Kilic Gunes et al. (2025) applied convolutional neural networks (CNNs) and random survival forests (RSF) to peripheral blood smear and flow cytometry data, achieving over 95% accuracy for AML detection and a concordance index (C-index) above 0.77 for prognostic risk stratification, thereby facilitating earlier diagnosis in resource-limited settings [38]. Thirugnanasambandam et al. (2025) implemented Random Forest models and neural networks using clinical and mutation profiles to predict relapse and guide personalized therapeutic decisions [51]. Li et al. (2025) employed XGBoost coupled with SHAP-based explainability on biomarker data to predict 30-day mortality in high-risk patients [52]. In lymphoma, Carreras et al. (2024) applied CNNs with transfer learning on histopathology images for subtype classification, while Uppal et al. (2025) leveraged ResNet and DenseNet architectures on whole-slide images to achieve precise differentiation among lymphoma categories [53,54]. Carreras et al. (2022) further combined ensemble survival ML models with genomic and clinical variables to estimate patient outcomes [55]. In multiple myeloma, Eweje et al. (2021) integrated CNNs with radiomic features extracted from bone marrow imaging to detect lesions and developed DL ensemble approaches that predict treatment response and survival based on biomarker profiles [56]. Table 2 summarizes hematological malignancies studies, highlighting different AI applications and their associated clinical outcomes.

Collectively, these advances significantly enhance diagnostic accuracy, prognostic assessment, and precision-medicine strategies in hematologic malignancies.

## 4. Enhancing Trial Efficiency Through ctDNA in Hematologic Malignancies

**Figure 3:**
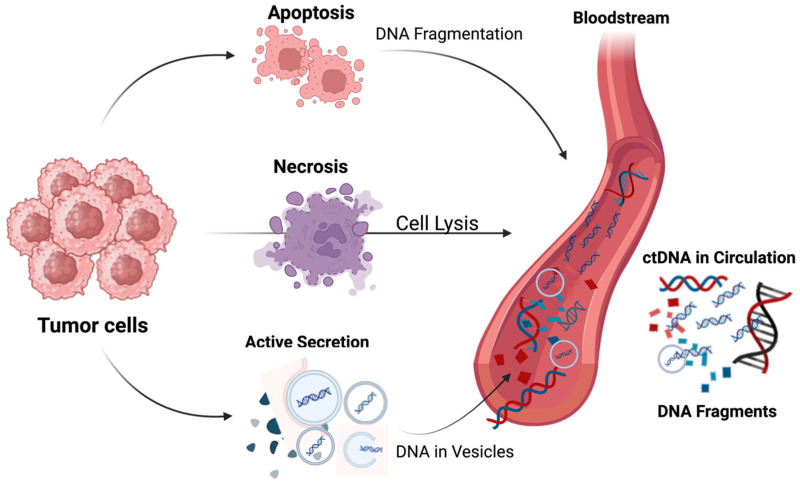
Mechanisms of circulating tumor DNA (ctDNA) release and detection. Tumor cells release fragmented DNA into the bloodstream through apoptosis, necrosis, and active secretion via extracellular vesicles. These DNA fragments enter the circulation, where they mix with cell-free DNA from normal cells and can be isolated from blood samples for molecular analysis. Analysis of ctDNA enables non-invasive assessment of tumor-specific genetic alterations and disease dynamics (created in BioRender. t, J. Mahmoud. A.M. (2026) https://BioRender.com/4hy22h1, accessed on 26 January 2026).

As an emerging field, ctDNA is increasingly recognized as a non-invasive biomarker with substantial clinical value in hematologic malignancies [66]. ctDNA represents the proportion of cell-free DNA that originates from malignant cells, released into the bloodstream through apoptosis, necrosis or active secretion (Figure 3) [67,68,69,70].

Studies have shown that ctDNA contains clinically relevant mutations, reflects disease burden, and can detect therapy-resistant clones that may not be identified by conventional tissue biopsies [70]. Moreover, ctDNA dynamics correlate with treatment response, the early detection of relapse, prognosis, and estimation of drug resistance, and can support MRD monitoring, targeted therapy selection, and immunotherapy surveillance [71,72,73,74]. For example, ctDNA characterization identified markers of relapse and refractoriness in mantle cell lymphoma (MCL), demonstrating its potential utility in capturing tumor heterogeneity, particularly alterations involving the BTK gene [75,76].

Liquid biopsy is a well-established method for the analysis of ctDNA [77,78]. However, it still requires standardization due to limited sensitivity and variability in tumor-specific analysis methods [79,80,81]. Recent technologies, including next-generation sequencing (NGS), duplex sequencing, phased variant detection, and droplet digital PCR (ddPCR), have expanded the capability to detect tumor DNA in plasma or serum, rendering ctDNA analysis increasingly clinically actionable [80,82,83]. Beyond its role in traditional diagnostics, ctDNA offers multidimensional and quantitative information that can be integrated into trials to enhance adaptive decision-making, improve outcomes assessment, and increase trial efficiency [84].

Real-time tracking of disease progression using ctDNA plays a very crucial role in hematologic malignancies, as it can identify molecular recurrence at earlier stages than conventional imaging modalities such as positron emission tomography (PET)/CT [85]. Prediction of residual metabolic disease activity followed by MRD assessment, represents a key treatment milestone at the end of therapy that allows for the design of more streamlined clinical trials [85,86,87,88]. Modern basket trials in hematologic malignancies increasingly integrate multi-omics biomarkers to guide treatment strategies. Moreover, adaptive trial designs are incorporating real-time longitudinal omics data to dynamically adjust treatment and dosing [89]. In clinical trials, ctDNA can be employed to select patients based on the presence of specific molecular alterations for enrolment, to stratify study populations into high-risk and low-risk cohorts, and to monitor response to anticancer treatments [90]. A comprehensive overview of clinical trials in hematologic malignancies employing ctDNA-based approaches to guide therapeutic interventions is presented in Table 3, highlighting the transition toward molecularly driven trial designs.

Recently, in patients with peripheral T-cell lymphoma (PTCL), ctDNA analysis revealed that high pretreatment ctDNA burden and recurrent mutations, particularly *TP53*, *STAT3*, *TET2*, *DNMT3A*, and *RHOA*, were associated with poor survival outcomes and high relapse rates. Notably, end-of-treatment (EOT) MRD negativity was rare, even among patients achieving complete or partial remission, and persistent MRD strongly predicted inferior progression-free and overall survival [91,92,93,94].

## 5. Focusing on Real-World Evidence

In hematologic malignancies, RWE offers a complementary perspective to inform optimal study design, elucidate treatment algorithms, and the personalization of patient care beyond the scope of standard RCTs [95,96,97]. RWE derived from real-world data (RWD), which includes electronic health records, disease registries, insurance claims databases, treatment assignment systems, tumor genomic profiling records, and patient outcome reports, provides a valuable complement to traditional RCT evidence [95,97,98,99]. These prospective cohort studies, which evaluate interventions in routine clinical settings while retaining randomization, are non-randomized interventional studies that follow prospective protocols without random allocation. In contrast, RCTs may employ external control arms constructed from RWD to augment or replace traditional randomized comparators [100].

Within the digital health data system, EHRs contain routine patient records generated by healthcare providers [101]. Clinical decision support tools and structured data reports are utilities within EHRs that help users identify high-risk drug allergy labels and correct or remove inaccurate or outdated entries in the patient’s chart [102]. Comprehensive health insurance claims databases, as a source of RWE, allow investigation of geographic variations (regional differences) in key metrics such as disease incidence, patient demographics, treatment modalities (e.g., surgery, ablation, systemic therapy), and potentially clinical outcomes [103,104,105].

RWE is particularly valuable in this setting as it can provide evidence on treatment efficacy and safety across broader patient populations. It can help detect rare adverse events and inform lifelong patient care, as well as facilitate regulatory decision-making and post-marketing surveillance [106].

For example, RWE indicates that Carfilzomib, Lenalidomide, and Dexamethasone (KRd) in relapsed/refractory multiple myeloma have an acceptable safety profile and effective clinical outcomes with a median progression-free survival (PFS) of 26 months and a 2-year overall survival (OS) rate of 70% [107]. A US-based real-world study of Chimeric antigen receptor T (CAR-T) cell therapy evaluated treatment patterns and clinical outcomes following CAR-T failure or relapse. Results showed that 44.7% of patients initiated subsequent treatment at a median of 263 days post-CAR T infusion, with systemic chemotherapy representing the most commonly used conventional approach [108].

For trials in which enrolling the randomized control groups is challenging, such as those involving rare subtypes and high-risk populations, real world data can be used to construct synthetic or external control arms (ECAs). These ECAs provide a comparator in single-arm or early-phase studies [109,110,111]. Recent data suggest that RWD-derived ECAs may have great potential, particularly when traditional RCTs are not feasible in certain hematologic settings [109,112,113]. However, synthetic control arms have some limitations, including bias due to unmeasured confounders; this bias could potentially be overcome by using careful cohort selection, covariate harmonization and sensitivity analyses. Selukar et al. described a blinatumomab case study in which investigators accounted for potential unmeasured confounders, such as the different index dates for the trial and external cohort, namely the time of blinatumomab initiation in study and the start of the last salvage therapy in the external dataset. The author sought to reduce discrepancies in disease classification and response assessment by harmonizing both datasets through standardized, shared data definitions [114]. Another investigator proposed a (Partial Bias Correction) PBC approach to mitigating bias due to unmeasured covariates by limiting the maximum tolerable amount of bias and calibrating the trade-off between bias and variance when borrowing strength from SC. In reality, statisticians and clinical partners may find it difficult to decide the largest acceptable bias [115]. While data collection is an integral part of research and drug development, post-approval therapies face ongoing regulatory safety concerns. In this context, RWE offers a pathway for post-marketing surveillance and long-term safety monitoring [116,117,118].

## 6. Regulatory Considerations

### 6.1. Artificial Intelligence: Approval, Safety, and Liability (FDA and EU AI Act)

A central legal and regulatory challenge associated with the use of AI in healthcare concerns the liability for errors, adverse outcomes and negative consequences. AI networks and systems, particularly those based on machine learning, are trained on large sets of data and they evolve dynamically over time, making their behavior difficult to fully predict in all clinical scenarios, which may lead to inappropriate diagnostic interpretations or treatment recommendations, raising concerns regarding patient safety, accountability, and regulatory oversight [119,120]. The examination of the legal and regulatory framework has led to the emergence of definitions for both synthetic data and RWD. At present, the EU Data Governance Act (DGA) is the primary legal instrument that explicitly references synthetic data. Following recent developments in the EU AI Act, synthetic data are increasingly associated with “general-purpose AI models”, accompanied by specific requirements regarding risk management, transparency, and methodology [121].

### 6.2. ctDNA: Diagnostic Tests, Clinical Validation

Gene profiling technologies provide a distinct view into tumor biology although they are accompanied by significant challenges in biomarker discovery, analysis, and validation. These challenges include technical variability, interindividual heterogeneity and the critical requirement for standardization. Such limitations can impact the reliability and reproducibility of study results, frequently delaying regulatory approval and hindering the translation of biomarkers into routine clinical practice [89]. In clinical trials, ctDNA MRD is increasingly used to select and stratify patients. MRD positivity is closely correlated with poor clinical outcomes—an opportunity for earlier therapeutic intervention in cancer management [122]. The U.S. Food and Drug Administration has already approved a series of ctDNA-based companion diagnostic tests, supporting the analytical validation of ctDNA as a biomarker for regulatory use in early-stage solid tumors. There are several MRD detection methods such as the tumor-informed method, panel-based next-generation sequencing (NGS), tumor-naïve methods or a reduced candidate gene panel [123]. ctDNA might be influenced by the histology, grade, stage, or size of the tumor so that the timing for this measurement would need to be established in consultation with the FDA during the submission process and supported by the performance characteristics of the study test relative to disease characteristics and tumor biology. The variability of results from ctDNA assays can potentially be reduced by the adoption of standardized pre-analytical protocols, through stringent analytical validation with clearly established detection limits, use of error-corrected sequencing technologies (unique molecular identifiers, UMIs), the harmonization bioinformatics pipelines and participation in external quality control programs based on regulatory and consensus recommendations [123,124,125,126].

### 6.3. RWE: Data Privacy, HIPAA/GDPR, Quality Standards

RWE may be used to evaluate the effectiveness and safety of treatments; however, its application in hematology remains carefully regulated, with relatively few approvals by the U.S Food and Drug Administration (FDA) or the European Medicines Agency (EMA) relying primarily on RWE. Key challenges include variable data quality, insufficient sample sizes, unmeasured confounding, missing data and heterogeneous endpoint definitions, all of which contribute to an increased risk of bias [127]. Secondary uses of health data are subject to stringent safeguards aimed at minimizing re-identification risk and protecting patient privacy. In the United States, such use is governed by the Health Insurance Portability and Accountability Act (HIPAA), while in the European Union, it is regulated under the General Data Protection Regulation (GDPR) [128,129,130,131].

## 7. Ethical Considerations

### 7.1. Artificial Intelligence: Bias, Explainability, Accountability, and Equitable Access

In healthcare, the introduction of biased AI systems raises fundamental ethical concerns related to justice, non-maleficence and patient autonomy. Algorithmic bias can disproportionately affect underrepresented populations, while limited explainability may hinder informed consent and shared decision-making, as clinicians and patients may struggle to interpret AI-generated recommendations. These ethical considerations can undermine patient confidence and clinical care and compromise decision-making. Consequently, the development and implementation of AI in healthcare must prioritized transparency, accountability and equity to ensure fair and responsible clinical adoption [120,132]. There may be an unintended effect on patient autonomy with AI. The opacity of a subset of AI algorithms can conceal rationales and affect autonomy and the quality of informed consent. It also may influence autonomy by limiting choice, as in deciding about human- vs. AI-led health care treatment decisions. Building out systems and recommendations for transparent reporting and organized AI model evaluation can improve both the credibility and ethical deployment of AI in healthcare [133,134,135].

### 7.2. ctDNA: Consent, Genetic Findings, Clinical Utility

Access to genomic and multi-omics data can contribute to precision medicine by enabling highly individualized, “custom-fit” therapeutic strategies. However, it also raises significant ethical and privacy concerns, as misuse or unauthorized disclosure of such data can compromise patient confidentiality. Germline genomic information which remains stable throughout an individual’s lifetime, constitutes a uniquely identifying biometric, and its exposure can pose substantial privacy risks [89,136]. Pathologists and laboratory professionals must develop a deep under-standing of the many policy dimensions that influence the molecular diagnostic considerations because they drive molecular diagnostics: their availability and accessibility. Attention needs to be paid during training to the responsible use of genomic data, laying out for trainees what are their ethical responsibilities as custodians of patient confidentiality and transparent participants in testing [137,138,139].

### 7.3. RWE: EHR-Derived Data, Patient Consent, Re-Identification Risk

Practical barriers, such as feasibility, data governance and long-term sustainability often restrict timely access to multi-national RWD due to diverse legal and ethical considerations, with patient consent and privacy protection frequently resulting in delays in achieving data availability, thereby constraining the ability to generate timely safety evidence and inform regulatory and clinical decision-making [140,141]. Indeed, many of these data can be obtained as part of standard clinical care rather than research and patients may not be aware that they are being used in a secondary capacity—eroding traditional informed consent paradigms and emphasizing the rationale for transparent, broad or dynamic consent strategies. Whilst datasets often are de-identified, developments in data linkage and genomics increase the risk that patients could be re-identified and suffer stigma, discrimination or psychological harm, particularly in rare hematologic diseases [142,143,144,145].

## 8. Limitations in Current Implementation

In practice, the implementation of AI, ctDNA, and RWE continues to face substantial difficulties that limit their effectiveness. The limitations encompass suboptimal data quality and interoperability; algorithmic biases, dataset shift, and constrained generalizability; opaque “black-box” models undermining clinician confidence; and unclear, evolving regulatory expectations for validation and monitoring. Contemporary research emphasizes multicenter prospective AI trials incorporating clinical endpoints, the creation of explainable and fairness-conscious models, and risk-stratified regulatory structures to facilitate AI integration throughout the clinical trial continuum [57,146,147]. In the context of ctDNA, the limitations involve biological heterogeneity in tumor shedding, insufficient sensitivity for low tumor burden detection, inconsistencies between circulating and tissue-based genotyping, and extensive variability in assays and bioinformatics workflows that obstruct standardization and threshold establishment. Future research trajectories involve expansive interventional studies assessing ctDNA-directed escalation/de-escalation, global harmonization of pre-analytical procedures and sequencing/bioinformatics workflows, and integrative multi-modal methodologies (e.g., fragment omics, methylation analysis, long-read sequencing, and ML signatures) to augment sensitivity and specificity [148,149,150,151]. The core limitations of RWE include heterogeneous EHR and claims data, issues with data missingness and miscoding, inconsistent capture methods, and serious risks of confounding, selection, and information biases that compromise causal inference. Current efforts prioritize robust, auditable real-world data (RWD) platforms; refined causal inference and trial design strategies (including external control arms, pragmatic studies, and hybrid trial-RWD integrations); and extensive application of AI/large language models to automate data abstraction and cleaning, thereby generating regulator-grade real-world evidence (RWE) for policy and practice [152,153,154,155].

## 9. Integration of AI, ctDNA, and RWE: A Combined Approach to Trial Design

**Figure 4:**
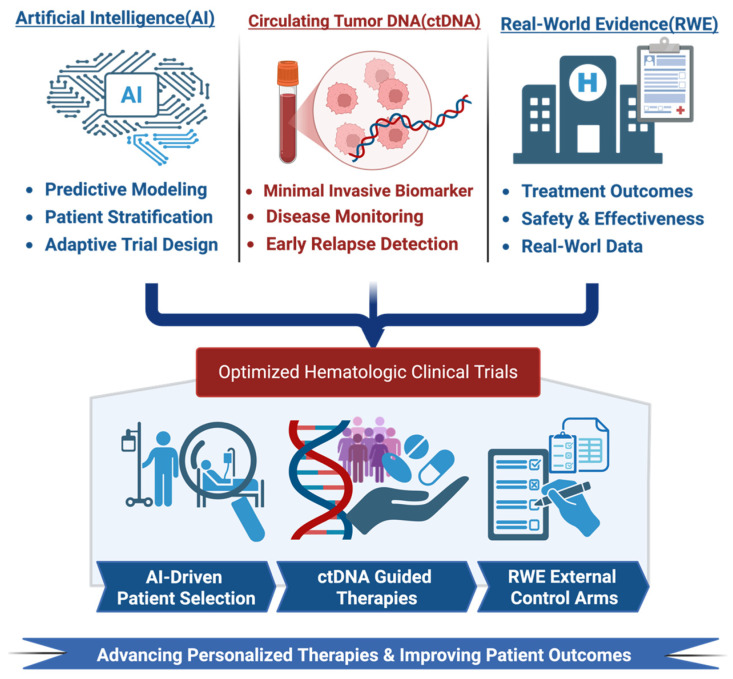
Integrated framework illustrating how artificial intelligence (AI), circulating tumor DNA (ctDNA), and real-world evidence (RWE) jointly enhance hematologic clinical trials. AI enables predictive modeling, patient stratification, and adaptive trial design; ctDNA provides a minimally invasive biomarker for disease monitoring and early relapse detection; and RWE contributes complementary data on treatment outcomes, safety, and effectiveness. Their integration supports optimized trial design through AI-driven patient selection, ctDNA-guided therapeutic decisions, and RWE-based external control arms, ultimately advancing personalized therapies and improving patient outcomes (created in BioRender. t, J. Mahmoud. A.M. (2026) https://BioRender.com/9sszq40, accessed on 26 January 2026).

While AI, ctDNA, and RWE each provide significant advancements independently, their integration creates a synergistic framework that enables a paradigm shift in clinical trial design. Rather than functioning as parallel tools, these components interact dynamically, as AI leverages ctDNA-derived molecular data to refine predictive models, while RWE continuously validates and recalibrates these models in real-world populations. This interconnected system transforms clinical trials into adaptive, feedback-driven ecosystems. The combination of AI, ctDNA, and RWE is propelling a novel era of hematologic clinical trial designs characterized by increased efficiency, adaptability, and being effectively patient-centric. This paradigm aligns the precision hematology transition from rigid, tumor-type based protocols toward biomarker-driven, master protocol designs, including basket, umbrella, and adaptive platform trials [3,156,157,158,159]. AI platforms now interrogate expansive clinicogenomic databases, EHRs, and structured master protocol datasets to inform nearly every aspect of clinical trial design. These tools are being used to refine eligibility criteria, identify biomarker-enriched cohorts, select sensitive endpoints, and determine realistic sample sizes, while also discovering predictive biomarkers thereby enhancing the probability of detecting therapeutic benefit [1,3,4,156,157,158,160]. Across multiple precision oncology programs, AI-facilitated matching of therapies to molecular profiles has been associated with better outcomes compared with non-matched treatment approaches, highlighting the importance of optimized clinical trial design (Figure 4) [3,156,158].

ctDNA provides a complementary capability as minimally invasive tool for real-time assessment of MRD, tumor burden, and therapeutic response. Across diverse hematological malignancies and solid tumor settings, ctDNA-based MRD predicts relapse months before standard imaging and strongly enriches for high-risk populations [8,122,161,162,163]. This capability has enabled ctDNA-guided adjuvant trials include CIRCULATE-Japan, which incorporates concurrent escalation (intensified therapy for ctDNA-positive patients) and de- escalation (reduced or omitted chemotherapy for ctDNA-negative patients) arms, within a single adaptive platform [8,161]. Similar ctDNA-informed designs have been implemented in lung cancer and other settings, allowing earlier endpoint assessment, shortening trial duration, and minimizing patient exposure to ineffective therapies [122,161,163,164]. For instance, AI-enriched ctDNA analysis has achieved mutation-detection limits as low as 0.002% allele fraction, with relapse detection sensitivities above 90% and lead times of several months compared to conventional imaging. These advances have the potential to redefine how disease progression and treatment response are assessed in clinical trials [6,161,163].

Meanwhile, RWE derived from routine practice EHRs, registries, and master-registry trials offers the longitudinal depth and scale that traditional trials often lack. These data are increasingly used to develop external or hybrid control arms, refine inclusion and exclusion criteria, and ensure that biomarker-selected trials populations more closely resemble the diverse patients treated in real-world clinical settings [1,3,156,158,161]. When coupled with advanced NLP and large language models, radiology reports, and pathology descriptions can be converted into quantifiable variables for trial design and post-marketing surveillance [1,160,165]. Importantly, the integration of these modalities enables a continuous learning loop in which clinical trial data, molecular disease monitoring, and real-world outcomes are iteratively fed back into AI systems. This approach not only enhances trial efficiency but also supports real-time optimization of therapeutic strategies, ultimately improving patient outcomes.

This synergistic framework integrating AI, ctDNA, and RWE can seamlessly support adaptive platform trials, tumor-agonistic basket studies, N-of-1 designs, and ctDNA- guided adjuvant trials [3,8,157,158,159,161]. In an adaptive system, ctDNA monitoring combined with AI-based risk stratification can govern decisions regarding treatment escalation, de-escalation, or discontinuation. In N-of-1 and basket paradigms, multimodal models can identify the most promising targeted or immunotherapy combination tailored for an individual patient’s molecular profile [3,156,157,158,164]. As hematology trials widely embed routine molecular profiling and post-treatment ctDNA surveillance, and feed their results back into learning health system registries, clinical research is beginning to resemble a continuous feedback loop rather than a series of isolated experiments [3,156,158,159,161]. In this emerging model, trials evolve from drug-centric evaluations of average efficacy toward patient-oriented learning systems that continuously update hypotheses using real-time molecular dynamics and real-world evidence.

## 10. Future Perspective in Clinical Trials Design

This approach—AI, ctDNA, and RWE—is expected to fundamentally reshape hematologic clinical trial methodology in the coming years. Recent work also emphasizes that RWE still struggles at some stages with data quality and interpretability, but AI has the capacity to significantly improve RWD utilization, draw causal inferences, and connect the two approaches to routine clinical practice and trial evidence accumulation [155,166]. Using AI mathematical modeling, we are capable of simulating product performance, predicting the interaction of product attributes with patient physiology and clinical outcomes, tailoring treatments, and advancing personalized medicine. This approach will increasingly support predictive modeling, reinforcement learning and Bayesian optimization will drive adaptive trial designs that dynamically modify enrollment criteria [167,168,169,170].

The lack of benefit with intrathecal or high-dose methotrexate prophylaxis for prevention of CNS relapse in DLBCL among high-risk patients has not been confirmed in large retrospective studies, but it may be more targeted to treat occult disease in patients with detectable ctDNA at baseline [171]. In the future, ctDNA detection technologies are expected to be increasingly integrated into new therapeutic strategies to dynamically monitor changes in tumor burden, disease progression, and prognosis [172]. Classic clinical variables such as age, serum lactate dehydrogenase (LDH), Eastern Cooperative Oncology Group (ECOG), performance status, and extranodal involvement retain robust and independent prognostic significance within AI-based models regardless of model complexity or data integration strategy [173]. Given the high cost of novel drug development, RWD are anticipated to play an increasingly central role in regulatory decision-making and value assessment [5]. One of the most promising developments is the emergence of “self-learning trials,” in which incoming clinical, molecular, and real-world data streams are iteratively incorporated to refine trial design, patient selection, and therapeutic interventions in near real-time. Such systems may reduce development timelines, improve endpoint sensitivity through ctDNA-based surrogate markers, and enhance regulatory decision-making through the integration of high-quality real-world data. However, realizing this vision requires overcoming key challenges, including data standardization, cross-platform interoperability, regulatory acceptance of adaptive AI systems, and ethical considerations related to data privacy and algorithmic bias. Addressing these challenges will be essential to fully harness the synergistic potential of these technologies and to translate innovation into clinical benefit.

Over the coming years, integrating AI, ctDNA and RWE into routine clinical practice will require not only technological refinement but also resolution of regulatory, clinical, and economic challenges. Trial frameworks are expected to leverage AI-driven algorithms for real-time treatment modifications based on integrated data streams, supporting more adaptive and personalized therapeutic strategies. AI-guided interpretation of ctDNA kinetics will allow earlier and better-informed identification of molecular relapse, with preliminary implications for adaptive treatment approaches and a reduction in sample size required to show benefit at the clinical level.

## 11. Conclusions

The integration of AI, ctDNA, and RWE is reshaping the design and conduct of hematologic clinical trials. Each of these components contributes distinct value: AI enables advanced data analysis and patient stratification, ctDNA provides real-time insights into disease dynamics, and RWE extends evidence generation to broader and more representative patient populations. The integration of AI, ctDNA, and RWE represents a coherent and evidence-based advancement of hematologic clinical trial design. Across hematologic malignancies, AI enables improved patient stratification, endpoint selection, and data integration from complex clinical and molecular sources, improving trial efficiency and interpretability while preserving methodological rigor [1,3,5]. ctDNA provides a validated, minimally invasive biomarker for molecular residual disease assessment, treatment response, monitoring, and early relapse detection, supporting adaptive and biomarker-driven trial frameworks with clinically meaningful surrogate endpoints [6,7,8,49,75,76,77,78]. Concomitantly, RWE complements randomized evidence by extending evaluation to broader, more representative patient populations, facilitating external control arms, post-marketing surveillance, and long-term safety assessment, particularly in rare or high-risk hematologic settings [85,96,99,100,101,102,103].

When combined, these approaches support more adaptive, efficient, and patient-centered clinical trial frameworks. This integrated model allows for improved trial design, earlier detection of treatment response or failure, and better alignment between experimental research and real-world clinical practice without compromising regulatory or ethical standards [3,10,119,120,121]. Despite these advances, several challenges remain, including data standardization, validation of novel endpoints such as ctDNA, management of bias in AI models, and regulatory and ethical considerations related to data use. Addressing these issues will be essential to ensure the safe and effective implementation of these technologies. Therefore, the successful implementation of this integrated approach depends on robust data quality, standardization, transparency, and regulatory oversight, as well as careful attention to privacy, bias, and equity considerations [107,108,110,117]. Overall, the convergence of AI, ctDNA, and RWE represents a promising step toward more precise, flexible, and clinically relevant research in hematologic malignancies. Continued efforts to integrate and refine these approaches will be critical to translating innovation into improved patient outcomes [5,15,119].

## Figures and Tables

**Table 1:** Hematological malignancies in AI-enabled clinical trials and conventional (non-AI) clinical trial dimensions.

Dimensions	AI-Enabled Clinical Trials	Conventional (Non-AI) Clinical Trials	Citations
Primary outcome success	70–81% of AI RCTs show improved primary endpoints vs. usual care or clinicians alone	RCTs employing traditional non-AI interventions have historically exhibited lower success rates.	[57,58,59]
Diagnostic accuracy	Frequent gains in detection rates and sensitivity (e.g., adenoma detection, seizure hours, risk stratification)	Baseline clinician or routine care performance	[57,58,59]
Designs used	Mostly parallel RCTs, often single-center; growing use in adaptive designs and risk-based monitoring	Conventional fixed trial designs predominate, while adaptive designs exhibit limited AI integration.	[57,58,60]
Trial timelines & efficiency	AI applications in trial recruitment, study design, and monitoring are associated with 30–50% faster trial completion times and potential cost reductions of up to 40%, as demonstrated in modeling and observational studies.	Traditional recruitment bottlenecks, more site visits, higher costs	[61,62]
Accuracy over timepoints	Numerous tools remain limited to single-timepoint analyses (e.g., imaging); the development of explicit multi-timepoint, context-aware models is recognized as a critical research priority.	Standard repeated measures analysis yet lacks true “algorithmic” integration of longitudinal data.	[57,63]

**Table 2:** Hematological malignancies with different AI applications and clinical outcomes.

Author and Published Year	AI Applications	Techniques	Data Types	General Output
**Leukemia**				
Kilic Gunes et al. (2025) [38]	Early detection, classification	CNNs, RSF, DL algorithms	Peripheral blood smears, CBC and multiparametric immunophenotyping of circulating blood cells	AML detection from blood images (>95% accuracy/sensitivity); risk stratification (concordance index > 0.77); automated smear analysis for early diagnosis.
Thirugnanasambandam et al. (2025) [51]	Prognosis & risk prediction	RF, Neural Networks	Clinical laboratory parameters, mutation profiles	Predicts relapse risk, survival, supports personalized therapy.
Li et al. (2025) [52]	Predicting complications	XGBoost, LightGBM, SHAP analysis	Integrated clinical variables with multiplex molecular or protein biomarker panels	Predict 30-day mortality and high-risk patients in leukemia + infections.
**Lymphoma**				
Carreras et al. (2024) [53]	Subtype classification& diagnosis	DL (CNN, transfer learning), interpretable ML	Histopathology images, immune-oncology panels	Automated subtype classification and feature extraction.
Uppal et al. (2025) [54]	Histopathology image detection	Transfer learning models (VGG, ResNet, DenseNet)	High-resolution whole-slide image	Distinguish between lymphoma subtypes with high diagnostic accuracy.
Carreras et al. (2022) [55]	Survival & outcome	Ensemble ML, Survival ML	Structured clinical data, standard laboratory tests, and genomic alternation data.	Support risk stratification and treatment decisions.
**Multiple myeloma**				
Eweje et al. (2021) [56]	Lesion detection & diagnosis	CNN, Radiomics + ML	Bone marrow aspirate/biopsy morphology, tissue histology, and radiologic imaging	Detects myeloma bone lesions and morphological features.
Romero et al. 2024 [64]	Prognosis & survival prediction	DL, Ensemble ML	Clinical variables, imaging, biomarkers	Predicts response to therapies, survival outcomes.
Allegra et al. 2022 [65]	Clinical decision support	Advanced ML, NLP (extracting data)	EHR with multi-omics layers	Supports personalized treatment planning and trial matching.

ML, Machine Learning; RF, Random Forest; CNN, Convolutional Neural Network; XGBoost, eXtreme Gradient Boosting; VGG, Visual Geometry Group; NLP, Natural Language Processing; EHR, Electronic Health Record; LightGBM, Light Gradient Boosting Machine; ResNet, Residual Network; DenseNet, Densely Connected Network.

**Table 3:** Comprehensive overview of clinical trials in hematologic malignancies employing ctDNA-based approaches to guide therapeutic interventions, highlighting the transition toward molecularly driven trial designs.

Identifier	Agent	Method	Phase	Condition	Enrollment	Use of ctDNA
NCT04604067	Acalabrutinib	PET/ctDNA-guided escalation	II	DLBCL	260	Treatment-guiding biomarker + MRD response marker
NCT03758989	R-CHOP	FDG-PET + ctDNA	II	DLBCL	40	Disease response and risk-stratification biomarker
NCT06693830	standard-of-care chemotherapy with or W/O Rituximab	ctDNA PhasED-seq	Interventional	DLBCL	40	MRD biomarker used for treatment de-escalation
NCT04980222	Glofitamab + R-CHOP	ctDNA	II	DLBCL	46	Interim MRD biomarker guiding therapy escalation
NCT03311958	Nivolumab	ctDNA	I	DLBCL	15	MRD relapse surveillance
NCT04401774	Nivolumab	ctDNA	II	PCNSL	14	Molecular response monitoring
NCT06362148	Standard-of-care Treatment	NGS + ctDNA-ddPCR + PET/CT	Observational	PTCL	50	MRD surveillance
NCT06954805	R-EPOCH	ctDNA	II	PTLD	30	Guide treatment decisions
NCT03269669	Obinutuzumab	ctDNA	II	FL	73	Disease-monitoring biomarker
NCT06744075	N/A	ctDNA	Interventional	BCL	108	Lymphoma progression or death
NCT05255354	CAR-T therapy	ctDNA + clonoSEQ platform	Observational	DLBCL, MCL, and FL	300	MRD assessment
NCT04599634	VENOM	ctDNA	I	FL, MZL, MCL, CLL, and BCL	11	MRD assessment
NCT03706625	N/A	ctDNA	Observational	Non-Hodgkin Lymphoma	201	Diagnostic and prognostic biomarkers
NCT04062877	N/A	ctDNA	Observational	Lymphoma	60	Precise diagnosis and prognosis
NCT02612350	N/A	ctDNA	Observational	Mutation & Neoplasms	1106	Early detection of cancer
NCT03710603	D-VRd	cfDNA/ctDNA and PFS (NGS-based assays)	III	MM	709	Predict myeloma outcomes
NCT03421132	Standard-of-care Treatment	ctDNA	Observational	MM	96	Molecular profiling/mutation tracking for disease progression and survival
NCT04157569	Standard-of-care Treatment	ctDNA-ddPCR	Observational	AL	200	Exploratory liquid biopsy biomarker

cfDNA—Cell-Free DNA, PET/CT—Positron Emission Tomography/Computed Tomography, FDG-PET—Fluorodeoxyglucose Positron Emission Tomography, NGS—Next-Generation Sequencing, FL—Follicular Lymphoma, MZL—Marginal Zone Lymphoma, MCL—Mantle Cell Lymphoma, CLL—Chronic Lymphocytic Leukemia, BCL—B-cell lymphoma, DLBCL—Diffuse Large B-Cell Lymphoma cell, VENOM—Venetoclax With Obinutuzumab and Magrolimab, ddPCR—Droplet Digital Polymerase Chain Reaction.

## Data Availability

No new data were created or analyzed in this study.
